# Basic psychological needs, quality of motivation, and protective behavior intentions: a nationally representative survey study

**DOI:** 10.1080/21642850.2023.2257295

**Published:** 2023-09-25

**Authors:** Meri Pietilä, Kaisa Saurio, Frank Martela, Mia Silfver, Nelli Hankonen

**Affiliations:** a Faculty of Social Sciences, Tampere University, Tampere, Finland; b Faculty of Social Sciences, University of Helsinki, Helsinki, Finland; c Department of Industrial Engineering and Management, Aalto University, Aalto, Finland

**Keywords:** COVID-19, protective behaviors, self-determination theory, autonomous motivation, behavioral intention

## Abstract

**Objective:**

Building on the Self-Determination Theory, this study examines how basic psychological need satisfaction related to COVID-19 behavioral measures is associated with motivation quality and whether motivation quality is associated with intention to wear a face mask and to avoid meeting others.

**Methods:**

Cross-sectional survey study involving a nationally representative sample of Finnish adult population aged 18–79 (*N* = 2272, M age = 48.63, SD = 16.89, 975 men and 1297 women) was conducted in Finland in May 2021 when protective behaviors were recommended to prevent acceleration of the epidemic. Measures included scales of Basic Psychological Need Satisfaction in Adhering to COVID Prevention Measures, Motivation to Adhere to COVID Prevention Measures, Perceived Personal Risk, Fear of COVID-19, and Protective Behavior Intention. Analysis of variance tests, linear regression analysis, and multinomial logistic regression were conducted. Perceived personal risk and fear of COVID-19 were controlled for in the regression analyses.

**Results:**

All three psychological needs were positively related to autonomous motivation (all *p *< .001). Autonomous motivation (range *OR *= 1.82–3.55, *p = *.001) was consistently related to intention to wear a mask and intention to avoid meeting people. Controlled motivation (range *OR* = .66*–*.93, *p *= .001*–*.457) was associated with decreased protective behavior intentions. The effects of amotivation (range *OR* = .65*–*1.02, *p* = .001*–*.911) varied across analyses.

**Conclusions:**

Fostering autonomous motivation could increase adherence to protective behaviors in situations without clear mandates.

The management of large-scale public health crises such as a pandemic requires behavior changes from the citizens. The COVID-19 pandemic led governments and authorities around the globe mandating citizens to engage in protective behaviors, such as wearing a face mask and social distancing (Han et al., 2020; Prasiska et al., 2022). For example, the use of face masks and respirators was recommended by the World Health Organization (WHO) to effectively stop the spread of COVID-19 in communities (World Health Organization, 2021). Studies have found several factors to be associated with adherence to government guidelines during the COVID-19 pandemic (Woodland et al., 2022), such as trust in the healthcare system, risk perception, and fear of COVID-19 (Ahorsu et al., 2022; Alijanzadeh et al., 2021). Citizen’s volitional adherence is important, as the required behaviors need to be performed throughout individual’s everyday activities and are difficult to monitor and enforce (Martela et al., 2021). In understanding such voluntary compliance, the distinction between autonomous and controlled motivation as conceptualized by Self-Determination Theory (SDT) (Deci & Ryan, 2012; Ryan & Deci, 2017) is crucial. Across domains, including health-related behaviors (Ng et al., 2012), autonomous motivation – based on values, ownership, and interest – is associated with higher commitment and more sustained behavior change than controlled motivation – which is based on external pressure from rewards and punishments, or internal pressure from avoidance of guilt or shame (Hagger et al., 2020; Ryan & Deci, 2017). Autonomous motivation thus provides a potentially relevant way to improve adherence to guidelines. Several theoretical frameworks have been found to be viable for investigating protective behaviors, e.g. Health Behavior Model (Soltani et al., 2022) and Integrated Social Cognition Model (Lin et al., 2020). However, studies investigating Self-Determination Theory in this context have been sparse.

Autonomous motivation itself is fostered, according to SDT, by a social environment that satisfies basic psychological needs for autonomy, competence, and relatedness (Ryan & Deci, 2017; Vansteenkiste et al., 2020). In the context of adherence to protective behavior, autonomy means a sense of volition and internal locus of causality, meaning that the individuals have a sense of willingness and self-endorsement of one’s actions, competence means a sense of efficacy and effectance meaning that the individuals feel that they have the ability to adhere to the measures, and relatedness is about a sense of mutual care and belonging meaning that the individuals feel a sense of connection with others through adhering to the measures. Given previous research linking need satisfaction with autonomous motivation and need frustration with controlled motivation (Ntoumanis et al., 2021; Ryan & Deci, 2017) satisfaction of these needs could be highly relevant for motivation for voluntary compliance.

Autonomous motivation has consistently been found to predict COVID-19-related protective behaviors (Alivernini et al., 2021; Guay et al., 2021; Morbée et al., 2021), with somewhat inconsistent effect of controlled motivation (Morbée et al., 2021). Building on these studies, we examine whether autonomous motivation is associated with more consistent behavioral intentions in different situations, with a nationally representative sample. Besides, previous studies have not investigated the quality of motivation and basic psychological need satisfaction in protective behavior context. Thus this will be the first to introduce scales to measure need satisfaction in the context of behavioral adherence and examine their relation with autonomous motivation. Protective behavior adherence has been found to vary among demographic groups, such as age and gender (Liu & Arledge, 2022). However, most studies so far have been conducted with small samples and thus have a limited ability to reliably compare potential differences in demographic groups. Our research questions are: (1) How do demographic groups (gender, educational level, and age) differ in (a) basic psychological need satisfaction in adhering to the protective behaviors to stop the spread of the COVID-19, (b) motivation quality for protective behaviors? (2) Is basic psychological need satisfaction related to motivation quality? (3) Is there a difference in how autonomous motivation, controlled motivation, and amotivation are related to self-reported intention to use a face mask and to avoid meeting people in different situations? Based on SDT, we test the hypothesis that autonomous motivation is associated with stronger intentions than controlled motivation (pre-registered hypothesis at https://doi.org/10.17605/OSF.IO/JEYRV). We expect fear of COVID-19 and perceived personal risk to increase autonomous motivation and intention for protective behavior. Therefore, similarly to Morbée et al. (2021), these constructs are controlled for in our analyses.

## Method

### Participants

The survey was conducted in Finland in May 2021 by an independent company. Participants were invited from an online panel based on age, gender, and geographical location to be representative of the national population. Sample (*N* = 2272, M age = 48.63, SD = 16.89, 975 men and 1297 women) had a slight over-representation of women and respondents from capital region. Electronic informed consent was obtained from respondents at the beginning of the survey. At the time of the data collection, protective behaviors were recommended by the Finnish authorities to prevent acceleration of the epidemic (Finnish Institute for Health and Welfare, 2021). The study was reviewed by the University of Helsinki Ethical Review Board in Humanities and Social and Behavioral Sciences (15/2021).

### Measures

*Motivation to adhere to COVID prevention measures* was measured with a modified version from Morbée et al. (2021) (overall measures), translated to Finnish, with an adapted amotivation subscale (Markland & Tobin, 2004) embedded (see questionnaire in Supplement A). Exploratory factor analysis-based factor scores were used in the analyses (see Table 1), autonomous motivation *α* = .93, controlled motivation *α* = .72, amotivation *α* = .84. Two of the three items measuring introjected regulation did not load on the controlled but on the autonomous motivation factor instead (see also Discussion).

**Table 1.** Exploratory factor analysis on ‘motivation to adhere to COVID prevention measures’ scale.

Variable	Autonomous	Controlled	Amotivation	Communalities
Because I find them personally relevant	**.92**	–.11	.00	.87
Because they are in line with my values	.**87**	–.13	–.04	.85
Because I fully support them	.**86**	–.16	–.05	.84
Because I do it out of an obligation to myself	.**73**	.03	–.02	.55
Because then I can be proud of myself	.**70**	.12	.02	.47
Because I think they are important	.**56**	–.07	–.40	.81
Because I feel pressured to do so	–.20	.**80**	.08	.75
Because otherwise I will be criticized	–.00	.**69**	–.05	.47
Because I feel compelled to do so	–.12	.**65**	.16	.54
Because I would be ashamed if I didn’t do that	.34	.**48**	–.11	.37
I do not adhere to the measures because I do not see the point in them	–.07	–.04	.**85**	.80
I see no reason to adhere to the measures	.07	.00	.**81**	.58
I do not understand why I should adhere to the measures	–.00	.04	.**75**	.58
Initial Eigenvalue	6.33	2.20	1.06	
% of variance	48.68	16.93	8.16	

Note*.* Factor loadings over .40 appear in bold. *N* = 2272. The extraction method was maximal likelihood with an oblique (direct oblimin) rotation. The item stem was: Why do you adhere or would you adhere to measures to prevent the spread of the coronavirus (e.g. using a face mask, keeping a safe distance, and minimizing social contacts)? I adhere or would adhere to measures.

Based on questionnaires measuring *basic psychological need satisfaction* in other domains (Aelterman et al., 2016; Chen et al., 2015; Schultz et al., 2015), we formulated a broad list of need satisfaction items concerning adhering to COVID prevention measures. Together with a panel of SDT experts, we chose the most suitable six items (two per need) (see Table 2). To confirm that the respondents understood the items in the intended way, a small piloting was conducted (*n* = 5/ item) with questions inspired by Wolf et al. (2021), resulting in some optimizations of the wordings. *Protective behavior intention* when meeting people outside one’s household indoors and in a cafe, restaurant or bar indoors was measured with items developed by the authors concerning the intention to wear a face mask during one’s free time or to not attend these situations. Response options included intention to (1) wear a face mask part of the time, (2) wear a mask the whole time, (3) not wear a mask, and (4) not go at all. Three other measured situations had very skewed data distributions and thus excluded. *Perceived personal risk* variable was calculated by multiplying the perceived probability of infection by its perceived severity (Wolff et al., 2019), *α* = .50. The scale was developed by the authors and stems were ‘How likely do you think it is, that you will get a coronavirus infection in your free time in the next month, if you did nothing to protect yourself from it?’ and ‘If you would get a coronavirus infection, how serious a threat would you rate it to your health?’. *Fear of COVID-19* was measured on a scale from ‘does not scare me’ to ‘scares me’, with three items: ‘Spread of the coronavirus … ’, ‘That I would get infected myself … ’, and ‘That my close one would get infected … ’, formulated based on guide by WHO (2020) (*α* = .89).

**Table 2.** Conceptual links between basic psychological needs and principles for COVID-19 and other emergency communications.

Basic psychological need	Basic Psychological Need Satisfaction (BPNS –) items	Communication principles (Martela et al., 2021)
Autonomy	I feel that I have had freedom of choice in how I take action to prevent the spread of the coronavirus I feel that by following the recommendations to prevent the spread of the coronavirus, I have been able to promote values that are important to me	Provide a meaningful rationale Treat people as responsible agents Use non-controlling, informational language Appeal to the aspirations, goals, and values of the people Within necessary limits, provide choice on how to adhere to the rules
Competence	I feel that I have excellent skills to take action to prevent the spread of the coronavirus I feel that I have been able to act skillfully in accordance with the recommendations to prevent the spread of the coronavirus	Provide concrete instructions, clear expectations, and formulate collective goals to strive for Provide constructive, clear, and relevant feedback on how successful people have been in adherence to the measures Address key obstacles for change
Relatedness	I feel that by working to prevent the spread of the coronavirus, I have been able to show concern for others and others for me. I experience cohesion with others as I follow recommendations to prevent the spread of the coronavirus	Acknowledge people’s own perspectives, feelings, and potential conflicts Emphasize and facilitate shared identity and common fate Build trust through transparent and open communication Identify trusted messengers to mediate the guidelines to various groups Appeal to people’s natural willingness to help each other

Note*.* The stem was: The authorities have recommended several measures to prevent the spread of the coronavirus. How have you experienced measures this spring, for example using a face mask, keeping a safety distance, and minimizing social contacts?

### Statistical analyses

Research question 1 was investigated with one-way analysis of variance tests. Bootstrapping was conducted, and Welch’s *F* is reported. Games–Howell correction procedure was used for the post-hoc tests, due to unequal group sizes and variances (Field, 2018). Research question 2 was investigated in linear regression analysis, controlling for perceived personal risk and fear of COVID-19. Research question 3 was examined in a set of multinomial logistic regression analyses, controlling for perceived personal risk and fear of COVID-19 (see Supplement B1 for Bivariate Pearson correlations for variables in regression analyses). Data were analyzed using SPSS version 28. Unlike mentioned in the pre-registration, Spearman correlation was not used, as the independent variables were treated as continuous.

## Ethics statement

This study was performed in line with the principles of the Declaration of Helsinki. The study was reviewed by the University of Helsinki Ethical Review Board in Humanities and Social and Behavioral Sciences (15/2021).

## Results

### Demographic differences

There were statistically significant difference between women and men in basic psychological need satisfaction (autonomy *F*(1, 2076.07) = 34.16, *p *< .001, *η2 = *0.02, 95% CI[0.007, 0.026]; competence *F*(1, 1936.56) = 93.67, *p *< .001, *η2 = *0.04, 95% CI[0.027, 0.058]; relatedness *F*(1, 1925.37) = 84.81, *p *< .001, *η2 = *0.04, 95% CI[0.024, 0.054]) and autonomous motivation (*F*(1, 1886.76) = 88.69, *p *< .001, *η2 = *0.04, 95% CI[0.025, 0.056]), women having higher means in need satisfaction and autonomous motivation (Supplement B2).

Need satisfaction and autonomous motivation were significantly different between education levels (autonomy *F*(2, 406.217) = 6.21, *p *= .002, *η2 = *0.01, 95% CI[0.001, 0.013]; competence *F*(2, 408.08) = 10.36, *p *< .001, *η2 = *0.01, 95% CI[0.003, 0.019]; relatedness *F*(2, 407.59) = 7.58, *p *< .001, *η2 = *0.01, 95% CI[0.002, 0.015]) and autonomous motivation (*F*(2, 400.54) = 5.65, *p *= .004, *η2 = *0.01, 95% CI[0.001, 0.12]). Post-hoc tests showed statistically significant differences between upper secondary education and higher education in autonomy satisfaction (*p *= .003) and autonomous motivation (*p *= .002). In addition, bootstrapped comparison between basic education and upper secondary education in autonomy satisfaction was significant (95% CI[.028, .356]), although the difference was small. Differences in satisfaction of competence and relatedness between upper secondary education and higher education were statistically significant (both *p *< .001). Other comparisons were not statistically significant.

There were statistically significant differences between age groups in satisfaction of needs (autonomy *F*(3, 1249.37) = 50.24, *p *< .001, *η2 = *0.06, 95% CI[0.040, 0.077]; competence *F*(3, 2250.98) = 11.17, *p *< .001, *η2 = *0.02, 95% CI[0.006, 0.025]; relatedness *F*(3, 1248.49) = 54.53, *p *< .001, *η2 = *0.06, 95% CI[0.044, 0.082]) and autonomous motivation (*F*(3, 1249.69) = 90.61, *p *< .001, *η2 = *0.10, 95% CI[0.074, 0.119]). Older age groups had higher means in BPNS and autonomous motivation. Post-hoc tests showed statistically significant differences in autonomous motivation and satisfaction of autonomy and relatedness between all age groups (all *p *< .001), except between 18–34 and 35–49 year-olds in autonomous motivation (*p *= .576) and relatedness (*p *= .468). However, bootstrapped comparison in autonomy satisfaction was significant between 18–34 and 35–49 year-olds (95% CI[–.22592, –.00170]). Differences in satisfaction of competence were significant between age groups 18–34 and 50–64 (*p *< .001), 18–34 and 65–79 (*p *< .001), 35–49 and 65–79 (*p *= .012), and between 18–34 and 35–49 in bootstrapped comparison (95% CI[–.20663, –.02350]).

### Association of psychological need satisfaction and autonomous motivation

Results from hierarchical linear regression model with all BPNS and control variables showed that all variables had positive associations with autonomous motivation to adhere to protective behavior (Supplement C1): satisfaction of autonomy (standardized beta, *β = *.234, *p *< .001, 95% CI for B[.204, .271]) and relatedness (*β*I *= *.402, *p *< .001, 95% CI for B[.331, .396]) had larger effects than competence (*β = *.091, *p *< .001, 95% CI for B[.077, .150]).

### Associations of quality of motivation and protective behavior intention

Multinomial logistic regression analyses were performed to ascertain the effects of autonomous motivation, controlled motivation, amotivation, perceived personal risk, fear of COVID-19 and protective behavior intention when meeting people outside one’s household indoors (Table 3) and in a restaurant setting (Supplement E1). Separate analyses assessing relationships between the individual independent variables and the protective behavior intentions are reported in supplement D. Groups of intention to wear a mask the whole time, intention to wear a mask part of the time and intention to not go were compared to the reference category of intention to not wear a mask.

**Table 3.** Multinomial logistic regressions of associations between motivation qualities and categories of protective behavior intention when meeting others.

Reference category: Do not intend to wear a mask		B (SE)	*p*	Odds ratio (OR)	95% CI for OR
Intend, whole time	Intercept	−0.80 (.23)	<.001		
	Autonomous	0.96 (.11)	<.001	2.61	[2.11, 3.22]
	Controlled	−0.31 (.07)	<.001	0.73	[.64, .84]
	Amotivation	0.01 (.11)	.911	1.01	[.81, 1.27]
	Risk perception	0.02 (.01)	<.001	1.02	[1.01, 1.04]
	Fear	0.03 (.05)	.547	1.03	[.93, 1.14]
Intend, part of the time	Intercept	−0.19 (.21)	.379		
	Autonomous	0.34 (.09)	<.001	1.40	[1.17, 1.68]
	Controlled	−0.08 (.06)	.233	0.93	[.82, 1.05]
	Amotivation	−0.30 (.10)	.002	0.74	[.62, .90]
	Risk perception	0.02 (.01)	.013	1.02	[1.00, 1.03]
	Fear	−0.04 (.05)	.441	.96	[.87, 1.06]
Do not intend to go	Intercept	−1.30 (.28)	<.001		
	Autonomous	0.63 (.13)	<.001	1.87	[1.45, 2.41]
	Controlled	−0.41 (.09)	<.001	0.66	[.56, .79]
	Amotivation	0.02 (.13)	.857	1.02	[.80, 1.31]
	Risk perception	0.02 (.01)	.013	1.02	[1.00, 1.04]
	Fear	0.01 (.06)	.942	1.01	[.89, 1.14]

Note*. N* = 2272. Qualities of motivation were entered in the model simultaneously with the control variables of perceived personal risk and fear of COVID-19.

Note*. R2 *= 0.17 (Cox–Snell), 0.19 (Nagelkerke). Model *χ^2^*(15) = 431.19, *p* < 0.001.

Assessing protective behavior intention when meeting people outside one’s household indoors showed that increase in autonomous motivation was associated with increase in odds of intentions to wear a face mask the whole time and to not go, whereas controlled motivation was associated with decrease in the odds and amotivation was not statistically significant. Autonomous motivation was associated with increase in odds of intention to wear a mask for a part of the time, whereas controlled motivation was not statistically significant and amotivation was associated with decrease in odds.

Similar to the model above, model assessing intention in restaurant setting improved with the addition of the independent variables, *χ^2^*(15) = 619.76, Nagelkerke *R^2^ *= 0.24, *p *< .001. As autonomous motivation increased, odds of intention to wear a mask the whole time (*OR* = 3.55, 95% CI [2.58, 4.88], *p *< .001), part of the time (*OR* = 1.40, 95% CI [1.09, 1.78], *p *= .002), and to not go (*OR* = 1.82, 95% CI [1.42, 2.35], *p *< .001) increased. Increase in controlled motivation was associated with decrease in odds of intention to not go (*OR *= 0.80, 95% CI [0.68, 0.94], *p* = .008), but associations with intention to wear a mask the whole time and part of the time were not statistically significant. As amotivation increased, odds of intentions to wear a mask part of the time (*OR *= 0.41, 95% CI [0.33, 0.52], *p* < .001) and to not go (*OR *= 0.65, 95% CI [0.53, 0.81], *p *< .001) decreased. Association with intention to wear a mask the whole time was not statistically significant.

## Discussion

As expected, based on theory and previous studies (Alivernini et al., 2021; Guay et al., 2021; Morbée et al., 2021), autonomous motivation towards protective behaviors was positively associated with intentions of better *quality* of behavior, i.e. intending to wear a mask the whole time, and avoiding the situations altogether. In line with the SDT, need satisfaction was associated with autonomous motivation. The effects of demographic differences in levels of need satisfaction and autonomous motivation were mainly small, except for age: compared to the younger age groups, the oldest group reported higher autonomous motivation, and higher satisfaction of basic psychological needs in adhering to the protective behaviors. This may be due to older age groups also objectively being more at risk for severe COVID-19, and therefore finding the recommendations more meaningful to follow. Also, in relatedness need satisfaction, the older groups likely felt more connected to others in terms of having the sense of being protected by others (‘I have been able to show concern for others and others for me’). However, all age groups felt similarly competent in protective behaviors. Due to the age-linked severity of the COVID-19 pandemic, similar age-related patterning of the levels of these SDT concepts may be found in other countries, however, more research is needed before drawing conclusions on generalizability.

The factor structure of motivational regulations differed from the theory, with two items of approach component of ‘introjected regulation’ loading on autonomous, not controlled motivation. Also, in Guay et al. (2021) the avoidant form of controlled motivation negatively predicted social distancing, unlike the approach form. As suggested by Morbée et al. (2021), it would be beneficial to increase the number of items measuring introjected regulation to measure approach and avoidant components separately.

The data collection needed to be carried out urgently as we aimed to investigate protective behaviors during a pandemic situation in which rapid changes, such as an unexpected end to the pandemic, were possible. At the time of data collection, there were no thoroughly validated measures available for the constructs we intended to examine. Therefore, we developed new measures, the validity of which could not be thoroughly tested. Although a strength of the intention scale was measuring situations separately, its limitation was including two different behaviors in the response options. The option of not intending to go at all to the situation was interpreted as social distancing and it had similar associations with the independent variables as the category of intending to wear a mask the whole time. This is an interpretation, social distancing was not explicitly mentioned in the item. Also, this study examined intention, which may not lead to behavior. Additionally, BPNS subscales contained two items each. Future studies would benefit from increased number of items. It must be noted that all causal inferences made based on the results are on uncertain grounds, as this cross-sectional study was unable to test them rigorously. It is also possible that not all relevant confounders have been controlled for.

This study, using a large sample, presented a novel application of the basic psychological needs scale and examined SDT constructs in a less-researched context. The findings were in line with SDT, implying that it could be a viable theoretical starting point for investigating psychological well-being and motivation in emerging health crises.

## Supplementary Material

Supplemental Material

## Data Availability

The data is available for research, teaching and study at the Finnish Social Science Data Archive (https://urn.fi/urn:nbn:fi:fsd:T-FSD3597).

## Open Scholarship

This article has earned the Center for Open Science badges for Open Data and Open Materials through Open Practices Disclosure. The data and materials are openly accessible at https://services.fsd.tuni.fi/catalogue/FSD3597?lang=en&study_language=fi.

## Code availability

Analysis code is available on OSF: https://osf.io/nshd2/?view_only=None.

## Geolocation information

All data used in the article was collected in Finland.
