# Supplementary material for: Basic psychological needs, quality of motivation, and protective behavior intentions: a nationally representative survey study
Source: Health Psychol Behav Med. 2023 Sep 25;11(1):2257295. doi: 10.1080/21642850.2023.2257295 (PMC10557624; doi:10.1080/21642850.2023.2257295)
Supplement: Supplemental Material [file RHPB_A_2257295_SM9165.docx]

**Basic Psychological Needs, Quality of Motivation, and Protective Behavior Intentions: A Nationally Representative Survey** **Study**

Health Psychology and Behavioral Medicine

Meri Pietilä, Kaisa Saurio, Frank Martela, Mia Silfver, Nelli Hankonen

Correspondence: Meri Pietilä, Tampere University, [meri.pietila@tuni.fi](mailto:meri.pietila@tuni.fi)

Electronic Supplementary Material

**ELECTRONIC SUPPLEMENTARY MATERIAL A**

**Questionnaire**

| English version of the questionnaire by theoretical construct | Questionnaire in Finnish |
| --- | --- |
| Protective Behavior Intention | |
| If in the next 7 days you go to visit the following indoor spaces and there are people outside your household, are you intending to wear a mask?  Grocery store or other store  Bus, train, or other means of public transport  Meeting people outside your household indoors  Cafe, restaurant, or bar indoors  Indoor exercise  Scale  [ ] I Intend to wear a mask all the time  [ ] I intend to wear a mask part of the time  [ ] I do not intend to wear a mask  [ ] I do not intend to go at all | Jos tulevan 7 päivän aikana menet käymään seuraavissa sisätiloissa ja paikalla on taloutesi ulkopuolisia ihmisiä, aiotko käyttää maskia?  Ruokakauppa tai muu kauppa  Bussi, juna tai muu julkinen kulkuväline  Taloutesi ulkopuolisten ihmisten tapaaminen sisätiloissa  Kahvilan, ravintolan tai baarin sisätila  Liikuntaharrastus sisätilassa  Asteikko  [ ] Aion käyttää maskia koko ajan  [ ] Aion käyttää maskia osan ajasta  [ ] En aio käyttää maskia  [ ] En aio mennä ollenkaan |

| Basic psychological need satisfaction in Adhering to COVID Prevention Measures | |
| --- | --- |
| Experience of measures to prevent the spread of coronavirus  The authorities have recommended several measures to prevent the spread of the coronavirus. How have you experienced measures this spring, for example using a face mask, keeping a safety distance, and minimizing social contacts? | Kokemus toimista koronaviruksen leviämisen estämiseksi   Viranomaiset ovat suositelleet useita toimia koronaviruksen leviämisen estämiseksi. Miten olet kuluvan kevään aikana kokenut toimet, esimerkiksi kasvomaskin käytön, turvavälin pitämisen ja sosiaalisten kontaktien minimoimisen? |
| I feel that I have had freedom of choice in how I take action to prevent the spread of the coronavirus  I feel that I have excellent skills to take action to prevent the spread of the coronavirus  I feel connected with others as I follow the recommendations to prevent the spread of the coronavirus  I feel that by following the recommendations to prevent the spread of the coronavirus, I have been able to promote values that are important to me.  I feel that I have been able to act skillfully in accordance with the recommendations to prevent the spread of the coronavirus  I feel that by acting to prevent the spread of the coronavirus, I have been able to show concern for others and others for me.  Scale [ ] Very much [ ] Quite a lot [ ] Some [ ] A little [ ] Not at all | Koen, että minulla on ollut valinnanvapautta siinä, kuinka toteutan toimia koronaviruksen leviämisen estämiseksi  Koen, että minulla on erinomaiset taidot toteuttaa toimia koronaviruksen leviämisen estämiseksi  Koen, yhteenkuuluvuutta muiden kanssa noudattaessani suosituksia koronaviruksen leviämisen estämiseksi  Koen, että noudattamalla koronaviruksen leviämisen estämiseksi annettuja suosituksia olen pystynyt edistämään itselleni tärkeitä arvoja  Koen, että olen pystynyt taitavasti toimimaan suositusten mukaisesti koronaviruksen leviämisen estämiseksi  Koen, että toimiessani koronaviruksen leviämisen estämiseksi olen voinut osoittaa välittämistä muita kohtaan ja muut minua kohtaan  Asteikko  [ ] Hyvin paljon  [ ] Melko paljon  [ ] Jonkin verran  [ ] Vähän [ ] En ollenkaan |
| Motivation to adhere to COVID prevention measures | |
| Why do you adhere or would you adhere to measures to prevent the spread of the coronavirus (e.g., using a face mask, keeping a safe distance, and minimizing social contacts)? I adhere or would adhere to measures  Not true at all [] [] [] [] [] Exactly true  [Integrated regulation]  I adhere to measures because they are in line with my values  [Identified regulation]  I adhere to measures because I think they are important  I adhere to measures because I fully support them  I adhere to measures because I find them personally relevant  [Inrojected regulation]  I adhere to measures because I would be ashamed if I didn’t do that  I adhere to measures because then I can be proud of myself  I adhere to measures because I do it out of an obligation to myself    [External regulation]  I adhere to measures because otherwise I will be criticized  I adhere to measures because I feel pressured to do so  I adhere to measures because I feel compelled to do so    [Amotivation]  I do not understand why I should adhere to the measures  I see no reason to adhere to the measures  I do not adhere to the measures because I do not see the point in them | Miksi sinä noudatat tai noudattaisit toimia koronaviruksen leviämisen estämiseksi (esim. kasvomaskin käyttö, turvavälin pitäminen ja sosiaalisten kontaktien minimoiminen)? Noudatan tai noudattaisin toimia,  Ei pidä lainkaan paikkaansa [] [] [] [] [] Pitää täysin paikkansa  Noudatan toimia, koska ne ovat linjassa arvojeni kanssa  Noudatan toimia, koska ne ovat mielestäni tärkeitä  Noudatan toimia, koska seison täysin näiden toimien takana  Noudatan toimia, koska pidän niitä henkilökohtaisesti merkityksellisinä    Noudatan toimia, koska minua hävettäisi, jos en toimisi niin  Noudatan toimia, koska voin sitten olla ylpeä itsestäni  Noudatan toimia, koska teen sen velvollisuudesta itseäni kohtaan    Noudatan toimia, koska muuten minua arvostellaan  Noudatan toimia, koska koen, että olen painostettu toimimaan niin  Noudatan toimia, koska koen, että olen pakotettu toimimaan niin   En ymmärrä, miksi minun pitäisi noudattaa toimia  En näe mitään syytä noudattaa toimia  En noudata toimia, koska en näe niissä järkeä |
| Perceived personal risk | |
| How likely do you think it is, that you will get a coronavirus infection in your free time in the next month, if you did nothing to protect yourself from it?    very unlikely [] [] [] [] [] [] [] very likely  [] I have it now | Kuinka todennäköisenä pidät sitä, että saisit koronavirustartunnan seuraavan kuukauden aikana vapaa-ajalla, jos et tekisi mitään suojautuaksesi siltä?     erittäin epätodennäköisenä [] [] [] [] [] [] []  erittäin todennäköisenä  [ ] minulla on se nyt |
| If you would get a coronavirus infection, how serious a threat would you rate it to your health?  not serious at all [] [] [] [] [] [] [] very serious | Jos saisit koronavirustartunnan, kuinka vakava uhka arvioisit sen olevan terveydellesi?  ei lainkaan vakava [] [] [] [] [] [] []  erittäin vakava |
| Fear of COVID-19 | |
| Spread of the coronavirus…  Does not scare me [] [] [] [] [] [] [] Scares me  That I would get infected myself ...  Does not scare me [] [] [] [] [] [] [] Scares me  That my close one would get infected  Does not scare me [] [] [] [] [] [] [] Scares me | Koronaviruksen leviäminen…  Ei pelota minua [] [] [] [] [] [] [] Pelottaa minua   Se että saisin itse tartunnan...   Ei pelota minua [] [] [] [] [] [] [] Pelottaa minua   Se että läheiseni saisi tartunnan  Ei pelota minua [] [] [] [] [] [] [] Pelottaa minua |

**ELECTRONIC SUPPLEMENTARY MATERIAL B**

**Descriptive statistics**

**Table B1**

*Bivariate Pearson correlations*

| Variable | 1 | 2 | 3 | 4 | 5 | 6 | 7 | 8 | 9 |
| --- | --- | --- | --- | --- | --- | --- | --- | --- | --- |
| 1. Autonomy satisfaction | *—* |  |  |  |  |  |  |  |  |
| 2.Competence satisfaction | -.555** | — |  |  |  |  |  |  |  |
| 3. Relatedness satisfaction | .698** | .604** | — |  |  |  |  |  |  |
| 4. Fear of COVID-19 | .427** | .319** | -.521** | — |  |  |  |  |  |
| 5. Perceived personal risk | .383** | .261** | .429** | .610** | — |  |  |  |  |
| 6.Autonomous motivation | .691** | .555** | .770** | .602** | .514** | — |  |  |  |
| 7. Controlled motivation | -.265** | -.145** | -.086** | -.031 | -.130** | -.112** | — |  |  |
| 8. Amotivation | -.554** | -.470** | -.648** | -.519** | -.419** | -.761** | .176** | — |  |
| 9.Intention, indoor meeting | -.158** | -.112** | -.207** | -.165** | -.169** | -.237** | .055** | .189** | — |
| 10.Intention, restaurant | -.068** | -.055** | -.123** | -.039 | -.051* | -.096** | -.059** | .089** | .230** |

**Table B2**

*Means and standard deviations of gender (woman/man), education levels in basic psychological need satisfaction and autonomous motivation for adhering to COVID-19 protective behaviors. (N = 2272).*

| Variable | Gender | | | | Education level | | | | | |
| --- | --- | --- | --- | --- | --- | --- | --- | --- | --- | --- |
|  | Woman (N=1297) | | Man (N=975) | | Basic education (N=151) | | Upper secondary education (N=757) | | Higher education (N=1364) | |
|  | *M* | *SD* | *M* | *SD* | *M* | *SD* | *M* | *SD* | *M* | *SD* |
| Autonomy satisfaction | 3.56 | 0.94 | 3.32 | 0.96 | 3.55 | 0.96 | 3.36 | 0.10 | 3.50 | 0.93 |
| Competence satisfaction | 4.13 | 0.72 | 3.81 | 0.82 | 3.95 | 0.75 | 3.89 | 0.85 | 4.06 | 0.74 |
| Relatedness satisfaction | 3.90 | 0.99 | 3.48 | 1.14 | 3.76 | 1.06 | 3.59 | 1.13 | 3.79 | 1.04 |
| Autonomous motivation | 0.17 | 0.88 | -0.22 | 1.05 | 0.04 | 1.03 | -0.10 | 1.03 | 0.51 | 0.93 |
| Variable | Age group | | | | | | | | | |
|  | 18–34 (N=615) | | 35–49 (N=550) | | 50–64 (N=616) | | 65–79 (N=491 | |  |  |
|  | *M* | *SD* | *M* | *SD* | *M* | *SD* | *M* | *SD* |  |  |
| Autonomy satisfaction | 3.20 | 1.00 | 3.31 | 0.96 | 3.56 | 0.92 | 3.81 | 0.82 |  |  |
| Competence satisfaction | 3.86 | 0.82 | 3.97 | 0.79 | 4.05 | 0.76 | 4.12 | 0.73 |  |  |
| Relatedness satisfaction | 3.42 | 1.16 | 3.52 | 1.13 | 3.89 | 0.98 | 4.10 | 0.87 |  |  |
| Autonomous motivation | -0.32 | 1.02 | -0.24 | 1.02 | 0.17 | 0.88 | 0.45 | 0.74 |  |  |

**ELECTRONIC SUPPLEMENTARY MATERIALC**

**Linear regression analysis**

**Table C1**

*Hierarchical linear regression analysis. Predicting autonomous motivation with BPNS, perceived personal risk, and fear of COVID-19. (N = 2272)*

| Step | | B | SE | β | 95% CI | | p |
| --- | --- | --- | --- | --- | --- | --- | --- |
|  |  |  |  |  | LL | UL |  |
| 1 | Intercept | -3.160 | .065 |  | -3.287 | -3.034 | .000 |
|  | Autonomy | .283 | .018 | .278 | .247 | .319 | <.001 |
|  | Competence | .104 | .020 | .084 | .065 | .144 | <.001 |
|  | Relatedness | .475 | .017 | .525 | .441 | .508 | <.001 |
| 2 | Intercept | -3.155 | .062 |  | -3.276 | -3.034 | .000 |
|  | Autonomy | .248 | .018 | .243 | .213 | .282 | <.001 |
|  | Competence | .112 | .019 | .090 | .075 | .150 | <.001 |
|  | Relatedness | .415 | .017 | .459 | .382 | .448 | <.001 |
|  | Perceived personal risk | .017 | .001 | .200 | .014 | .019 | <.001 |
| 3 | Intercept | -3.360 | .062 |  | -3.481 | -3.239 | .000 |
|  | Autonomy | .238 | .017 | .234 | .204 | .271 | <.001 |
|  | Competence | .114 | .019 | .091 | .077 | .150 | <.001 |
|  | Relatedness | .364 | .017 | .402 | .331 | .396 | <.001 |
|  | Perceived personal risk | .009 | .001 | .108 | .007 | .011 | <.001 |
|  | Fear of COVID-19 | .121 | .010 | .198 | .102 | .140 | <.001 |

*Note.* R² = .642 for Step 1; R² = .675 for Step 2; R² = .696 for Step 3; ΔR² = .032 for Step 2; ΔR² = .021 for Step 3.

**ELECTRONIC SUPPLEMENTARY MATERIAL D**

**Multinomial Logistic Regression Analyses with Independent Variables in Separate Models**

**Table D1**

*Multinomial logistic regression models with motivation qualities, perceived personal risk, and fear of COVID-19 as individual predictors of protective behaviour intention when meeting people outside one’s household.*

| Reference category:   Do not intend to wear a mask | | B (SE) | Odds ratio (OR) | *p* | 95 % CI for OR |
| --- | --- | --- | --- | --- | --- |
| Intend, whole time | Intercept | -0.23 (.06) |  | <.001 |  |
|  | Autonomous | 1.18 (.08) | 3.26 | <.001 | [2.81, 3.78] |
| Intend, part of the time | Intercept | -0.07 (.06) |  | .205 |  |
|  | Autonomous | -0.60 (.06) | 1.82 | <.001 | [1.62, 2.04] |
| Do not intend to go | Intercept | -0.91 (.07) |  | <.001 |  |
|  | Autonomous | 0.78 (.09) | 2.19 | <.001 | [1.86, 2.58] |
| Intend, whole time | Intercept | -0.18 (.06) |  | <.001 |  |
|  | Controlled | -.040 (.06) | 0.67 | <.001 | [0.59, 0.76] |
| Intend, part of the time | Intercept | -0.17 (.06) |  | .002 |  |
|  | Controlled | -0.18 (.06) | 0.84 | .003 | [0.75, 0.94] |
| Do not intend to go | Intercept | -1.00 (.07) |  | <.001 |  |
|  | Controlled | -0.47 (.08) | 0.62 | <.001 | [0.53, 0.73] |
| Intend, whole time | Intercept | -0.23 (.06) |  | <.001 |  |
|  | Amotivation | -0.99 (.09) | 0.37 | <.001 | [0.31, 0.45] |
| Intend, part of the time | Intercept | -0.13 (.06) |  | .017 |  |
|  | Amotivation | -0.59 (.07) | 0.55 | <.001 | [0.49, 0.63] |
| Do not intend to go | Intercept | -0.95 (.07) |  | <.001 |  |
|  | Amotivation | -0.64 (.09) | 0.53 | <.001 | [0.44, 0.64] |

| Reference category:   Do not intend to wear a mask | | B (SE) | Odds ratio (OR) | *p* | 95 % CI for OR |
| --- | --- | --- | --- | --- | --- |
| Intend, whole time | Intercept | -1.35 (.15) |  | <.001 |  |
|  | Risk perception | 0.07 (.01) | 1.07 | <.001 | [1.05, 1.08] |
| Intend, part of the time | Intercept | -0.86 (.14) |  | <.001 |  |
|  | Risk perception | 0.04 (.01) | 1.04 | <.001 | [1.03, 1.06] |
| Do not intend to go | Intercept | -1.89 (.18) |  | <.001 |  |
|  | Risk perception | 0.05 (.01) | 1.05 | <.001 | [1.04, 1.07] |
| Intend, whole time | Intercept | -2.27 (.19) |  | <.001 |  |
|  | Fear | 0.45 (.04) | 1.56 | <.001 | [1.45, 1.68] |
| Intend, part of the time | Intercept | -1.26 (.17) |  | <.001 |  |
|  | Fear | 0.24 (.04) | 1.28 | <.001 | [1.19, 1.37] |
| Do not intend to go | Intercept | -2.44 (.23) |  | <.001 |  |
|  | Fear | 0.32 (.05) | 1.38 | <.001 | [1.26, 1.51] |

*Note.* N = 2272. Independent variables were investigated individually in separate models.

*Note*. Standard errors were corrected by using dispersion parameter based on deviance statistic in the model with risk perception as an independent variable.

*Note.* Box-Tidwell test indicated violation of the assumption of linearity of logit in models with autonomous motivation, perceived personal risk, and fear of COVID-19 as the independent variable.

**Table D2**

*Multinomial logistic regression models with motivation qualities, perceived personal risk, and fear of COVID-19 as individual predictors of protective behaviour intention in restaurant setting.*

| Reference category:   Do not intend to wear a mask | | B (SE) | *p* | Odds ratio (OR) | 95 % CI for OR |
| --- | --- | --- | --- | --- | --- |
| Intend, whole time | Intercept | 0.59 (.11) | <.001 |  |  |
|  | Autonomous | 1.91 (.12) | <.001 | 6.77 | [5.41, 8.49] |
| Intend, part of the time | Intercept | 1.75 (.10) | <.001 |  |  |
|  | Autonomous | 1.15 (.07) | <.001 | 3.15 | [2.73, 3.65] |
| Do not intend to go | Intercept | 1.61 (.10) | <.001 |  |  |
|  | Autonomous | 1.32 (.08) | <.001 | 3.73 | [3.20, 4.36] |
| Intend, whole time | Intercept | 0.20 (.08) | .012 |  |  |
|  | Controlled | -0.25 (.09) | .005 | 0.78 | [0.66, 0.93] |
| Intend, part of the time | Intercept | 1.15 (.07) | <.001 |  |  |
|  | Controlled | -0.07 (.07) | .351 | 0.93 | [0.81, 1.08] |
| Do not intend to go | Intercept | -1.02 (.07) | <.001 |  |  |
|  | Controlled | -0.33 (.08) | <.001 | 0.72 | [0.62, 0.83] |
| Intend, whole time | Intercept | 0.54 (.10) | <.001 |  |  |
|  | Amotivation | -1.46 (.12) | <.001 | 0.23 | [0.18, 0.30] |
| Intend, part of the time | Intercept | 1.56 (.09) | <.001 |  |  |
|  | Amotivation | -1.14 (.07) | <.001 | 0.32 | [0.28, 0.37] |
| Do not intend to go | Intercept | 1.44 (.09) | <.001 |  |  |
|  | Amotivation | -1.12 (.08) | <.001 | 0.33 | [0.28, 0.38] |

| Reference category:   Do not intend to wear a mask | | B (SE) | *p* | Odds ratio (OR) | 95 % CI for OR |
| --- | --- | --- | --- | --- | --- |
| Intend, whole time | Intercept | -1.73 (.24) | <.001 |  |  |
|  | Risk perception | 0.13 (.02) | <.001 | 1.14 | [1.10, 1.17] |
| Intend, part of the time | Intercept | -0.12 (.18) | .529 |  |  |
|  | Risk perception | 0.09 (.01) | <.001 | 1.10 | [1.07, 1.13] |
| Do not intend to go | Intercept | -0.49 (.19) | .010 |  |  |
|  | Risk perception | 0.11 (.01) | <.001 | 1.11 | [1.08, 1.14] |
| Intend, whole time | Intercept | -3.15 (.40) | <.001 |  |  |
|  | Fear | 0.79 (.09) | <.001 | 2.20 | [1.86, 2.62] |
| Intend, part of the time | Intercept | -1.18 (.28) | <.001 |  |  |
|  | Fear | 0.58 (.07) | <.001 | 1.79 | [1.56, 2.05] |
| Do not intend to go | Intercept | -1.79 (.30) | <.001 |  |  |
|  | Fear | 0.69 (.07) | <.001 | 1.98 | [1.72, 2.29] |

*Note.* N = 2272. Independent variables were investigated individually in separate models.

*Note*. Standard errors were corrected by using dispersion parameter based on Pearson statistic in the models with risk perception and fear of COVID-19 as independent variables.

*Note.* Box-Tidwell tests indicated violation of the assumption of linearity of logit in models with autonomous motivation, perceived personal risk, and fear of COVID-19 as the independent variable.

**ELECTRONIC SUPPLEMENTARY MATERIAL E**

**Multinomial Logistic Regression Analyses with Predictors Entered Simultaneously**

**Table** E1

*Multinomial logistic regressions of Associations Between Motivation Qualities and Categories of Protective Behaviour Intention in a Restaurant Setting*

| Reference category:   Do not intend to wear a mask | | B (SE) | *p* | Odds ratio (OR) | 95 % CI for OR |
| --- | --- | --- | --- | --- | --- |
| Intend, whole time | Intercept | -0.34 (.35) | .328 |  |  |
|  | Autonomous | 1.27 (.16) | <.001 | 3.55 | [2.58, 4.88] |
|  | Controlled | -0.08 (.10) | .457 | 0.93 | [0.75, .1.14] |
|  | Amotivation | -0.28 (.16) | .073 | 0.75 | [0.55, 1.03] |
|  | Risk perception | 0.03 (.01) | .066 | 1.01 | [1.01, 1.05] |
|  | Fear | 0.09 (.08) | .264 | 1.10 | [0.93, 1.29] |
| Intend, part of the time | Intercept | 1.05 (.30) | <.001 |  |  |
|  | Autonomous | 0.33 (.13) | .008 | 1.40 | [1.09, 1.78] |
|  | Controlled | 0.11 (.08) | .197 | 1.12 | [0.95, 1.32] |
|  | Amotivation | -0.89 (.12) | <.001 | 0.41 | [0.33, 0.52] |
|  | Risk perception | 0.01 (.01) | .344 | 1.01 | [0.99, 1.03] |
|  | Fear | 0.10 (.07) | .168 | 1.10 | [0.96, 1.27] |
| Do not intend to go | Intercept | -0.49 (.30) | .110 |  |  |
|  | Autonomous | 0.60 (.13) | <.001 | 1.82 | [1.42, 2.35] |
|  | Controlled | -0.23 (.09) | .008 | 0.80 | [0.68, 0.94] |
|  | Amotivation | -0.43 (.11) | <.001 | 0.65 | [0.53, 0.81] |
|  | Risk perception | 0.02 (.01) | .150 | 1.02 | [0.99, 1.04] |
|  | Fear | 0.18 (.07) | .013 | 1.19 | [1.04, 1.38] |

*Note.* N = 2272. Qualities of motivation were entered in the model simultaneously with the control variables of perceived personal risk and fear of COVID-19.

*Note.* *R2* = 0.24 (Cox–Snell), 0.26 (Nagelkerke). Model *χ2*(15) = 619.76, p < .001.*Note*. Box-Tidwell test indicated some violation of the assumption of linearity of the logit: interaction term of perceived personal risk and its log produced significant effect in predicting the category of intending to wear a mask the whole time (p = .000), and intending to wear a mask part of the time (p = .000), rather than not intending to wear a mask. Due to several predictors, this value was not possible to calculate for this interaction term for the category of not intending to go, rather than not intending to wear a mask.
